# High expression of AK1 predicts inferior prognosis in acute myeloid leukemia patients undergoing chemotherapy

**DOI:** 10.1042/BSR20200097

**Published:** 2020-06-17

**Authors:** Tong Qin, Hongmian Zhao, Yunli Shao, Ning Hu, Jinlong Shi, Lin Fu, Yijie Zhang

**Affiliations:** 1 Department of Hematology, Huaihe Hospital of Henan University, Kaifeng 475000, China; 2 Department of Hematology, The first Affiliated Hospital of Henan University, Kaifeng 475000, China; 3 Translational Medicine Center, Huaihe Hospital of Henan University, Kaifeng 475000, China; 4 Department of Biomedical Engineering, Chinese PLA General Hospital, Beijing 100853, China; 5 Department of Medical Big Data, Chinese PLA General Hospital, Beijing 100853, China; 6 Department of Hematology, The Second Affiliated Hospital of Guangzhou Medical University, Guangzhou 510260, China; 7 Translational Medicine Center, The Second Affiliated Hospital of Guangzhou Medical University, Guangzhou 510260, China; 8 Department of Medicine, Huaihe Hospital of Henan University, Kaifeng 475000, China

**Keywords:** acute myeloid leukemia, AK1, allogeneic hematopoietic stem cell transplantation, chemotherapy, prognosis

## Abstract

The purpose of the present study was to investigate whether expression levels of adenylate kinase 1 (AK1) were associated with prognosis of acute myeloid leukemia (AML) in patients treated with chemotherapy or allogeneic hematopoietic stem cell transplantation (allo-HSCT). 85 AML patients with AK1 expression report who received chemotherapy-alone and 71 who underwent allo-HSCT from The Cancer Genome Atlas database were identified and grouped into either AK1high or AK1low based on their AK1 expression level relative to the median. Then, overall survival (OS) and event-free survival (EFS) were compared between patients with high vs. low AK1 expression. In the chemotherapy group, high AK1 expression was favorable for both EFS (*P*=0.016) and OS (*P*=0.014). In the allo-HSCT group, there was no association for AK1 expression levels and clinical outcomes. Further analyses suggested that in the high AK1 expression group, EFS and OS were longer in patients treated with allo-HSCT compared with those treated with chemotherapy (*P*=0.0011; *P*<0.0001, respectively), whereas no significant differences were observed in the low AK1 expression group. In summary, we reported AK1 as an independent unfavorable prognostic factor of AML patients undergoing chemotherapy, and its use could also facilitate clinical decision-making in selecting treatment for AML patients. Patients with high AK1 expression may be recommended for early allo-HSCT.

## Introduction

Adenylate kinase is a small, usually monomeric, enzyme found in every living thing due to its crucial role in energetic metabolism [1]. Nine different adenylate kinase isoenzymes have been identified and characterized so far in human tissues, named AK1 to AK9 according to their order of discovery [2]. Adenylate kinase 1 (AK1) plays crucial roles in processes such as cellular phosphotransfer networks, neuronal maturation and regeneration, and myocardial energetic homeostasis [3–5]. The human AK1 gene contains several consensus p53 binding sites and plays a relevant role in the establishment of reversible cell-cycle arrests as induced by p53 in these cells [6]. Alterations in *TP53* was described in numerous cancer types including hematological neoplasms.

Acute myeloid leukemia (AML) is the most common acute leukemia in adults, which has a high degree of heterogeneity in clinical manifestations, cell morphology, cytogenetics and so on. Characterization of recurrent functional somatic mutations by target next-generation sequencing is helpful in identifying disease-associated mutations, which are more meaningful for clinical practice [7]. Such as *TP53* mutations was associated with poor outcome of AML patients [8]. However, the potential prognostic role and clinical implications of AK1 in AML remain unclear.

In the present study, we investigated whether the expression levels of AK1 could provide prognostic information on AML patients treated with chemotherapy or allogeneic hematopoietic stem cell transplantation (allo-HSCT), independently from a comprehensive panel of other established clinical and molecular predictors. Our findings suggest that AK1 may have future applications for guiding therapeutic interventions.

## Materials and methods

### Patients

**Figure 1:**
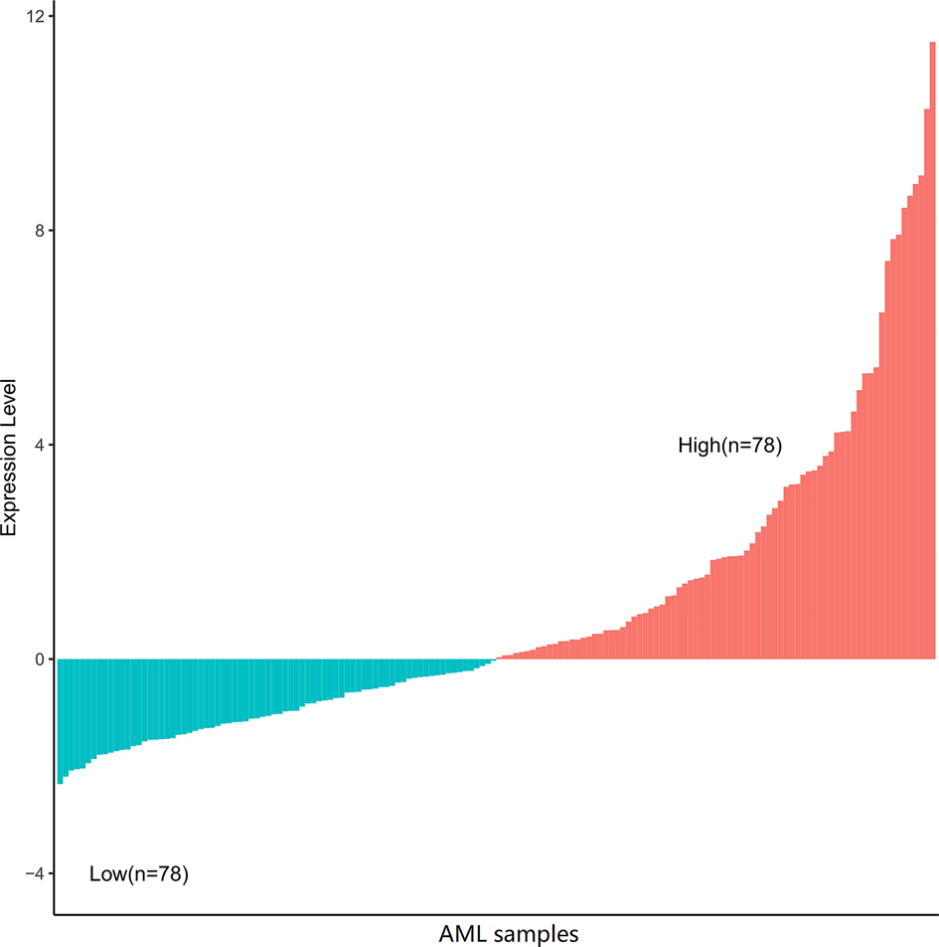
Expression level of AK1

Our study population consists of 156 patients aged 18–88 years received chemotherapy or allo-HSCT as consolidation for AML, during the time period between November 2001 and March 2010. The present study has been approved by Human Studies Committee of the Washington University. Patients with AML were included in a single center’s tissue protocol and followed NCCN guidelines to receive treatment. Patients with unfavorable risk underwent allo-HSCT if they were medically fit for the risks of transplantation, and if a suitably matched donor was available. Of the 156 patients, 85 were treated with chemotherapy-based consolidation, while remaining 71 patients were treated with allo-HSCT. Patients were selected from the research database if they had AK1 expression. We revealed that the expression level of AK1 changed when it was the median of the entire sequence (Figure 1). Then, who with expression levels higher than the median was defined as high expression group. The age, sex, peripheral white blood cell (WBC) counts, blast percentages of peripheral blood (PB) and marrow, French–American–British (FAB) subtypes, chromosome karyotype, and mutational status of recurrent genetic mutations were collected at diagnosis. All clinical data are available on the TCGA website.

### End points

Primary study end points were overall survival (OS) and event-free survival (EFS). OS was measured from the date of the patients were enrolled into the study until the date of death, and patients alive at last follow-up were censored. EFS was measured from the date of entry into the study to date of induction treatment failure, relapse from complete remission, or death resulting from any cause; patients not known to have any of these events at last follow-up were censored. All patients provided informed consent, and the study protocol was approved by the Washington University Human Studies Committee.

### Statistical analysis

The clinical and molecular characteristics of patients were reported using descriptive statistics. Numerical data were compared using Mann–Whitney *U*-test, and categorical data was compared using the Chi-square test and Fisher exact test. Survival was estimated according to the Kaplan–Meier method. The log-rank test was used for univariate comparisons. Cox regression was used to assess the association with a given variable with OS or EFS. Multivariate testing was performed using Cox proportional hazards models. The level of statistical significance was set as *P*<0.05. All statistical analyses were performed by SPSS software 20.0 and Graphpad Prism software 5.0.

## Results

### Comparison of characteristics between patients with high and low expression of AK1

Patients with AK1 expression levels above or equal to the median expression level were defined as high AK1 expression. The rest were defined as low AK1 expression. Median expression level for the chemotherapy group was 2.707 (range, 0.2269–14.0755). Median expression level for the allo-HSCT group was 2.2668 (range, 0.3664–9.9896). Comparison of clinical and molecular characteristics between two groups are shown in Table 1.

**Table 1:** Clinical and molecular characteristics of patients according to AK1 levels

Characteristics	Chemotherapy group			Allo-HSCT group		
	AK1^high^ (*n* = 43)	AK1^low^ (*n* = 42)	*P*	AK1^high^ (*n* = 36)	AK1^low^ *(n* = 35)	*P*
Age/years, median (range)	68 (33–88)	64 (22–81)	0.160*	54.5 (22–72)	46 (18–65)	0.011*
Age group/*n* (%)			0.587^§^			0.019^§^
<60 years	12 (27.9)	14 (33.3)		22 (61.1)	30 (85.7)	
≥60 years	31 (72.1)	28 (66.7)		14 (38.9)	5 (14.3)	
Gender/*n* (%)			0.591^§^			0.561^§^
Male	24 (55.8)	21 (50.0)		22 (61.1)	19 (53.4)	
Female	19 (44.2)	21 (50.0)		14 (38.9)	16 (45.7)	
WBC/×10^9^/l, median (range)	8.3 (0.7–171.9)	41.6 (1.0–297.4)	0.001*	10.4 (0.6–90.4)	34.2 (2.3–223.8)	0.001*
BM blast/%, median (range)	64 (30–97)	79 (32–99)	0.043*	71.5 (30–95)	70 (34–100)	0.401*
PB blast/%, median (range)	18 (0–91)	42.5 (0–98)	0.144*	41 (0–90)	58 (0–96)	0.091*
FAB subtypes/*n* (%)						
M0	6 (14.0)	1 (2.4)	0.052^§^	6 (16.7)	3 (8.6)	0.305^§^
M1	9 (20.9)	11 (26.2)	0.568^§^	15 (41.7)	8 (22.9)	0.090^§^
M2	11 (25.6)	10 (23.8)	0.850^§^	7 (19.4)	11 (31.4)	0.246^§^
M3	0 (0)	0 (0)		1 (2.8)	0 (0)	1.000^†^
M4	7 (16.3)	13 (31.0)	0.111^§^	3 (8.3)	10 (28.6)	0.027^§^
M5	6 (14.0)	7 (16.7)	0.728^§^	2 (5.6)	2 (5.7)	0.593^§^
M6	1 (2.3)	0 (0)	1.000^†^	1 (2.8)	0 (0)	1.000^†^
M7	2 (4.7)	0 (0)	0.494^†^	1 (2.8)	0 (0)	1.000^†^
Nc	1 (2.3)	0 (0)	1.000^†^	0 (0)	1 (2.9)	1.000^†^
Karyotype/*n* (%)						
t(8;21)(q22;q22.1)	1 (2.3)	5 (11.9)	0.085^§^	0 (0)	1 (2.9)	1.000^†^
inv(16)(p13.1q22) or t(16;16)(p13.1;q22)	0 (0)	6 (14.3)	0.012^†^	1 (2.8)	4 (11.4)	0.154^†^
Normal karyotype	18 (41.9)	22 (52.4)	0.331^§^	15 (41.7)	18 (51.4)	0.410^§^
t(9;11)(p21.3;q23.3)	1 (2.3)	0 (0.0)	0.494^†^	0 (0)	0 (0)	
t(v;11q23.3)	0 (0)	2 (4.8)	0.241^§^	3 (8.3)	0 (0)	0.239^†^
t(9;22)(q34.1;q11.2)	0 (0)	1 (2.4)	0.494^†^	2 (5.6)	0 (0)	0.493^†^
−5 or del(5q); −7; −17/abn(17p)	4 (9.3)	1 (2.4)	0.175^§^	2 (5.6)	3 (2.7)	0.620^§^
Complex karyotype	10 (23.3)	2 (4.8)	0.014^§^	10 (27.8)	1 (2.9)	0.004^§^
Others	8 (18.6)	2 (4.8)	0.048^§^	3 (8.3)	7 (20.0)	0.158^§^
Unknown	1 (2.3)	1 (2.4)	0.987^§^	0 (0)	1 (2.9)	1.000^†^
*FLT3-ITD/n* (%)			0.366^§^			0.368^§^
Presence	6 (14.0)	9(21.4)		7 (19.4)	10 (28.6)	
Absence	37 (86.0)	33 (78.6)		29 (80.6)	25 (71.4)	
*NPM1/n* (%)			0.215^§^			0.088^§^
Mutation	11 (25.6)	16 (38.1)		6 (16.7)	12 (34.3)	
Wild type	32 (74.4)	26 (61.9)		30 (83.3)	13 (65.7)	
*CEBPA/n* (%)			0.571^§^			0.022^§^
Single mutation	2 (4.7)	1 (2.4)		1 (2.8)	4 (11.4)	
Double mutation	0 (0)	0 (0)		0 (0)	3 (8.6)	
Wild type	41 (95.3)	41 (97.6)		35 (97.2)	28 (80.0)	
*RUNX1/n* (%)			0.147^§^			0.479^§^
Mutation	6 (14.0)	2 (4.8)		5 (13.9)	3 (8.6)	
Wild type	37 (86.0)	40 (95.2)		31 (86.1)	32 (91.4)	
*ASXL1/n* (%)			0.494^†^			1.000^†^
Mutation	2 (4.7)	0 (0)		1 (2.8)	0 (0)	
Wild type	41 (95.3)	42 (100)		35 (97.2)	35 (100)	
*TP53/n* (%)			0.115^§^			0.115^†^
Mutation	8 (18.6)	3 (7.1)		4 (11.1)	0 (0)	
Wild type	35 (81.4)	39 (92.9)		32 (88.9)	35 (100)	
*DNMT3A/n* (%)			0.859^§^			0.145^§^
Mutation	12 (27.9)	11 (26.2)		6 (16.7)	11 (31.4)	
Wild type	31 (72.1)	31 (73.8)		30 (83.3)	24 (68.6)	
*IDH1/IDH2/n* (%)						0.060^§^
Mutation	6 (14.0)	9 (21.4)	0.366^§^	12 (33.3)	5 (14.3)	
Wild type	37 (86.0)	33 (78.6)		24 (66.7)	30 (85.7)	
*TET2/n* (%)			0.115^§^			0.290^§^
Mutation	8(18.6)	3 (7.1)		1 (2.8)	3 (8.6)	
Wild type	35 (81.4)	39 (92.9)		35 (97.2)	32 (91.4)	

* Mann–Whitney *U-*test.

§ Chi-square test.

† Fisher exact test.

In chemotherapy-alone group, patients with high AK1 expression had a higher prevalence of complex karyotype and other karyotype, whereas the initial WBC, percentage of bone marrow (BM) blast, inv(16) or t(16;16) karyotype were lower. There were no significant differences between the two groups of age and gender distribution, PB blasts, FAB subtypes, *FLT3-ITD, NPM1, CEBPA, RUNX1, ASXL1, TP53, DNMT3A, IDH1/IDH2*, and *TET2* mutations.

In allo-HSCT group, patients with high AK1 expression tend to be older and more likely to have complex karyotype, whereas the initial WBC, FAB M4 subtype, and CEBPA mutations were lower. No significant differences were found in gender distribution, BM blast, PB blast, FAB subtypes other than M4 and frequent AML mutations (*FLT3-ITD, NPM1, RUNX1, ASXL1, TP53, DNMT3A, IDH1/IDH2*, and *TET2*).

### Univariate and multivariate Cox analysis for prognosis in the chemotherapy group and allo-HSCT group

To assess the prognostic significance of clinical and molecular variables, univariate and multivariate COX regression analyses were conducted, encompassing expression levels of AK1 (high vs. low), age (≥60 years vs. <60 years), peripheral WBC count (≥20 × 10^9^/l vs. <20 × 10^9^/l), *FLT3-ITD* (positive vs. negative), and frequent AML genetic mutations (*FLT3-ITD, NPM1, CEBPA, RUNX1, ASXL1, TP53, DNMT3A, IDH1/IDH2*, and *TET2*; mutated vs. wild type). The results of this analysis for the chemotherapy and allo-HSCT group were summarized in Tables 2 and 3.

**Table 2:** Univariate and multivariate analysis for EFS and OS based on chemotherapy

Variables	EFS		OS	
	HR (95%CI)	*P*-value	HR (95%CI)	*P*-value
Univariate analyses				
AK1 (high vs. low)	1.962 (1.220–3.153)	0.005	1.982 (1.228–3.199)	0.005
Age (≥60 vs. <60 years)	3.181 (1.769–5.719)	0.000	3.187 (1.752–5.797)	0.000
WBC (≥20 vs. <20×^9^/l)	1.029 (0.645–1.643)	0.903	1.007 (0.796–1.274)	0.953
*FLT3-ITD*	1.115 (0.611–2.037)	0.723	1.076 (0.589–1.966)	0.813
*NPM1* mutation	1.280 (0.782–2.095)	0.327	1.136 (0.689–1.874)	0.616
*TP53* mutation	3.011 (1.535–5.906)	0.001	2.952 (1.508–5.779)	0.002
*DNMT3A* mutation	1.702 (1.023–2.829)	0.040	1.632 (0.977–2.728)	0.062
*IDH1/IDH2* mutation	0.978 (0.544–1.758)	0.941	0.967 (0.715–1.308)	0.829
*TET2* mutation	0.823 (0.409–1.657)	0.586	0.733 (0.363–1.479)	0.386
*RUNX1* mutation	1.451 (0.692–3.042)	0.325	1.586 (0.755–3.333)	0.224
Multivariate analyses				
AK1 (high vs. low)	1.966 (1.136–3.400)	0.016	2.012 (1.151–3.518)	0.014
Age (≥60 vs. <60 years)	2.740 (1.480–5.072)	0.001	2.621 (1.389–4.949)	0.003
WBC (≥20 vs. <20×^9^/l)	1.496 (0.844–2.649)	0.168	1.220 (0.920–1.620)	0.168
*FLT3-ITD*	1.289 (0.655–2.535)	0.463	1.153 (0.574–2.317)	0.690
*NPM1* mutation	1.521 (0.806–2.872)	0.196	1.167 (0.626–2.177)	0.626
*TP53* mutation	2.900 (1.248–6.742)	0.013	2.315 (1.022–5.243)	0.044
*DNMT3A* mutation	1.668 (0.932–2.984)	0.085	1.740 (0.984–3.076)	0.057
*IDH1/IDH2* mutation	0.888 (0.461–1.713)	0.724	0.903 (0.664–1.267)	0.556
*TET2* mutation	0.832 (0.374–1.850)	0.652	0.584 (0.263–1.294)	0.185
*RUNX1* mutation	1.625 (0.681–3.880)	0.274	1.606 (0.680–3.794)	0.280

Abbreviations: CI, confidence interval; HR, hazard ratio.

**Table 3:** Univariate and multivariate analysis for EFS and OS based on allo-HSCT

Variables	EFS		OS	
	HR (95%CI)	*P*-value	HR (95%CI)	*P*-value
Univariate analyses				
AK1 (high vs. low)	1.038 (0.622–1.732)	0.888	1.136 (0.659–1.959)	0.646
Age (≥60 vs. <60 years)	1.101 (0.821–1.477)	0.522	0.843 (0.624–1.140)	0.268
WBC (≥20 vs. <20×^9^/l)	0.806 (0.621–1.046)	0.105	0.993 (0.754–1.308)	0.962
*FLT3-ITD*	0.737 (0.548–0.922)	0.044	0.775 (0.564–1.063)	0.114
*NPM1* mutation	1.102 (0.816–1.489)	0.525	1.115 (0.807–1.540)	0.510
*TP53* mutation	0.771 (0.461–1.290)	0.322	0.484 (0.281–0.834)	0.009
*DNMT3A* mutation	0.975 (0.721–1.317)	0.867	0.891 (0.649–1.224)	0.477
*IDH1/IDH2* mutation	1.184 (0.869–1.613)	0.285	1.129 (0.809–1.576)	0.475
*TET2* mutation	1.331 (0.743–2.383)	0.336	1.068 (0.596–1.914)	0.826
*RUNX1* mutation	0.841 (0.578–1.224)	0.366	0.641 (0.436–0.942)	0.024
Multivariate analyses				
AK1 (high vs. low)	1.141 (0.597–2.180)	0.689	0.973 (0.490–1.934)	0.938
Age (≥60 vs. <60 years)	1.043 (0.731–1.487)	0.818	0.875 (0.612–1.251)	0.464
WBC (≥20 vs. <20×^9^/l)	0.738 (0.536–1.016)	0.063	0.881 (0.626–1.238)	0.465
*FLT3-ITD*	0.651 (0.462–0.918)	0.014	0.670 (0.456–0.985)	0.042
*NPM1* mutation	1.261 (0.878–1.812)	0.209	1.166 (0.769–1.769)	0.469
*TP53* mutation	0.614 (0.339–1.111)	0.107	0.388 (0.205–0.733)	0.004
*DNMT3A* mutation	0.939 (0.665–1.325)	0.720	0.791 (0.549–1.140)	0.209
*IDH1/IDH2* mutation	1.056 (0.699–1.595)	0.797	1.147 (0.735–1.792)	0.545
*TET2* mutation	1.336 (0.719–2.485)	0.360	1.286 (0.674–2.453)	0.446
*RUNX1* mutation	0.736 (0.466–1.162)	0.188	0.538 (0.341–0.848)	0.008

Abbreviations: CI, confidence interval; HR, hazard ratio.

Univariate analysis indicated that with high expression of AK1, chemotherapy-alone patients had both shorter EFS (*P*=0.005) and OS (*P*=0.005) than those who with low expression of AK1. Age ≥ 60 years had an unfavorable effect on both EFS (*P*<0.001) and OS (*P*<0.001) for patients received chemotherapy alone. Mutations in *TP53* also contribute to poor EFS and OS in patients only received consolidation chemotherapy (*P*=0.001; *P*=0.002, respectively). *DNMT3A* mutations were associated with poor EFS (*P*=0.04) only. Based on multivariate analyses, high AK1 expression predicted a shorter EFS and OS independently (*P*=0.016; *P*=0.014, respectively), while Age ≥ 60 years indicated a relatively shorter EFS (*P*=0.001) and OS (*P*=0.003). *TP53* mutation status was also an independently risk factor of both EFS (*P*=0.013) and OS (*P*=0.044).

In the allo-HSCT group, univariate and multivariate analyses indicated that *FLT3-ITD* positive (*P*=0.044, 0.014) contributed to poor EFS, while *TP53* (*P*=0.009, 0.004) mutations and *RUNX1* (*P*=0.024, 0.008) mutations had unfavorable effect on OS. *FLT3-ITD* positive also significantly associated with shorter OS in multivariate analysis (*P*=0.042). However, AK1 had no effect on EFS and OS in univariate as well as multivariable analysis.

### Prognostic value of AK1 expression

**Figure 2:**
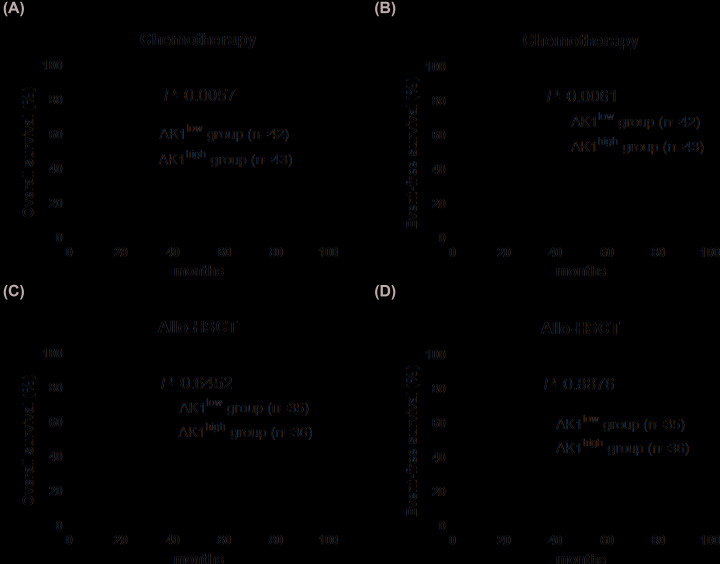
Kaplan–Meier curves of OS and EFS in different therapy groups (**A,B**) Chemotherapy-alone patients with high AK1 expression had shorter OS and EFS than those with low expression. (**C,D**) Expression of AK1 did not effect on survival after allo-HSCT.

**Figure 3:**
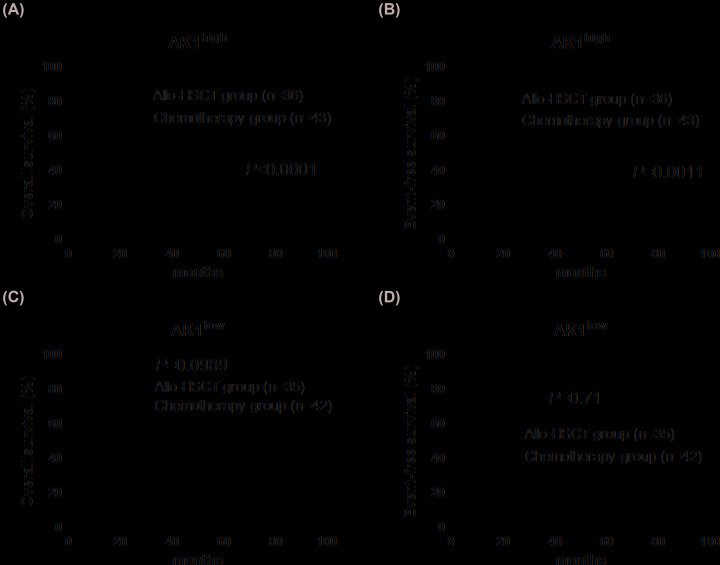
Kaplan–Meier curves of OS and EFS in different expression levels of AK1 (**A,B**) High AK1 expression patients had longer OS and EFS after allo-HSCT than chemotherapy-alone. (**C,D**) No difference in survival of patients with low expression of AK1 received allo-HSCT or chemotherapy.

The Kaplan–Meier survival estimate at the chemotherapy group reported a worse prognosis for both EFS (*P*=0.0061) and OS (*P*=0.0057) in patients with high-expression AK1 compared with those with low-expression (Figure 2A,B). In the allo-HSCT group, no significant differences were observed between patients with high vs. low AK1 expression (Figure 2C,D). We further divided all patients into two groups according to the expression levels of AK1. Kaplan–Meier survival estimate demonstrated that EFS (*P*=0.0011) and OS (*P*<0.0001) were longer in patients treated with allo-HSCT compared those treated with chemotherapy in the high AK1 expression group (Figure 3A,B); no significant differences were observed between patients treated with allo-HSCT and chemotherapy in the low AK1 expression group (Figure 3C,D).

### Associations between gene expression profiles and AK1 expression

**Figure 4:**
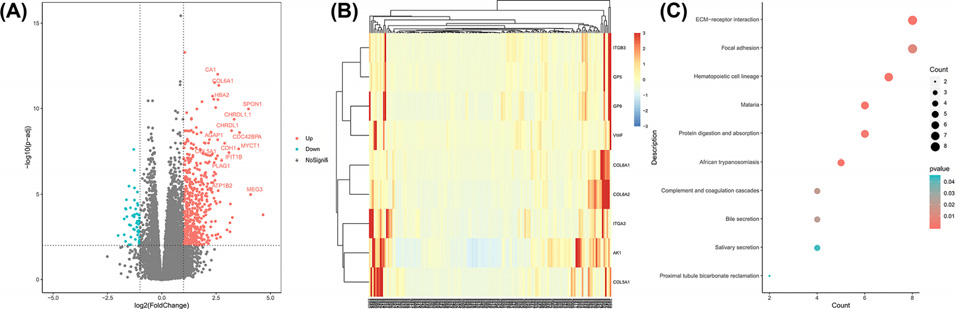
Genome-wide gene expression profile and cell signaling pathways associated with AK1 expression (**A**) Volcano plot of differential gene expression. AK1^high^ and AK1^low^ were marked by red and green circles, respectively. (**B**) Expression heatmap of associated with AK1 expression. (**C**) KEGG analysis of genes related to AK1 expression.

To further assess the role of AK1 in AML, we derived gene expression profiles by high throughput sequencing from TCGA data. We found 525 up-regulated and 53 down-regulated genes that were significantly associated with AK1 expression (*P*<0.01, fold change = 1, Figure 4A). These genes were presented in the aberrant expression heat map (Figure 4B). Further Kyoto Encyclopedia of Genes and Genomes (KEGG) enrichment analysis indicated that the genes associated with AK1 expression were mainly involved in “ECM−receptor interaction,” “focal adhesion,” “hematopoietic cell lineage,” “malaria,” “protein digestion and absorption,” “African trypanosomiasis,” “complement and coagulation cascades,” “bile secretion,” “salivary secretion,” and “proximal tubule bicarbonate reclamation” pathways (Figure 4C).

## Discussion

As current molecular stratification schemes do not fully grasp the heterogeneity of prognosis in patients with AML, the identification of novel prognostic markers is urgent. In the current study, higher AK1 expression presented worse survival prospects for AML patients who received chemotherapy. Additionally, the prognostic effect of AK1 expression may be overcome by allo-HSCT.

We showed that AK1 expression levels constitute independent prognostic marker of AML in a heterogeneous cohort administered chemotherapy. Both univariate analysis and multivariate analyses in the chemotherapy group indicated that high AK1 expression and *TP53* mutations are contribute to the poorer outcome of AML patients. These findings suggest that AK1 independently influences treatment outcomes and may synergistically drive leukemogenesis. More importantly, AK1 expression levels could be useful to the identification of patients with adverse outcome in AML patients administrated chemotherapy. Both univariate analysis and multivariate analyses indicated that AK1 expression levels have no effect on outcomes after allo-HSCT. However, the survival curves proved that high AK1 expressers administered allo-HSCT showed markedly improved OS and EFS in comparison with cases administered chemotherapy. In cases lowly expressing AK1, there was no advantage for those administered allo-HSCT in comparison with the chemotherapy group. These findings suggest that allo-HSCT overrides the prognostic ability of AK1 expression and patients with low AK1 expression may not benefit from allo-HSCT as first-line therapy. In the allo-HSCT group, *FLT3-ITD* and mutations in *TP53* and *RUNX1* all independently contributed to poor survival, which were consistent with previous research results that *FLT3-ITD* was associated with increased risk of relapse in AML, *RUNX1* mutations were independent predictor for inferior survival and *TP53* mutations adversely affect outcome in AML [8–10]. This indicated that allo-HSCT could not triumph over the adverse prognostic effect of them, which were consistent with previous research results that *FLT3-ITD, TP53*, and *RUNX1* mutations conferred a poor prognosis on AML even after allo-HSCT [11–13]. Hence, allo-HSCT is therefore considered a reasonable treatment option for patients with high AK1 expression.

Prominent features of complex karyotype cases are the frequent loss of 17p and/or TP53 gene mutation [14,15], occurring in approximately two-thirds of the cases, and a high prevalence of high-level DNA amplifications [15]. Complex karyotype has consistently been associated with a very poor outcome [16]. AML with inv(16) or t(16;16) has been associated with an improved relapse-free survival [17]. In general, the intensity and type of therapy are tailored according to the risk profile in AML, whereby chemotherapy is reasonable in favorable-risk AML and allo-HSCT is considered in adverse-risk AML [18–20]. Our data show that the adverse cytogenetic change complex karyotype appears more frequently in the high AK1 expression group, while favorable cytogenetic change inv(16)/t(16;16) tend to be observed in the low expression group. This implies that AK1 up-regulation may play the same role as complex karyotype in predicting prognosis for AML patients. Accordingly, down-regulation of AK1 may have similar prognostic features of inv(16)/t(16;16). The mechanisms concerning the regulation of AK1 expression and subsequent influence of AML treatment outcome remain to be elucidated.

AML in older patients generally had poorer prognosis due to higher mutation burden, poorer baseline performance status, and co-morbidities [21]. In our study, age ≥ 60 years had a negative impact on survival in the chemotherapy group, but it did not work in the allo-HSCT group. It suggested that age was an important prognostic factor of AML patients received chemotherapy only, and it might also be associated with fewer older patients received allo-HSCT.

There were certain limitations of the current study. The relatively small number of patients is the major limitation of our study. Certain genes were required to be deleted from the multivariate analysis due to their low mutation rate, in order to ensure statistical efficiency. In addition, the lack of original datum is the limitation of our retrospective analysis. In future work, we will conduct laboratory work to elucidate whether AK1 acts as a tumor promoter of AML and its underlying mechanisms.

In conclusion, our study provided new insight into risk stratification and the treatment selection of AML. AK1 expression could greatly contribute to the identification of patients with poor outcome in AML. Expression analysis of AK1 may be useful to improve the risk stratification of AML patients. AK1 was an independent prognostic factor for AML patients underwent chemotherapy. Allo-HSCT might be a better option for AML patients with high AK1 expression. Therefore, the expression analysis of AK1 may help identify cases in need of strategies to select the optimal treatment regimen between chemotherapy and allo-HCST. Because this was a non-randomized, retrospective observational study using registry data, which would allow for the introduction of bias. Larger studies may be warranted to further validate our results.

## Abbreviations

AK1: adenylate kinase 1
allo-HSCT: allogeneic hematopoietic stem cell transplantation
AML: acute myeloid leukemia
BM: bone marrow
EFS: event-free survival
FAB: French–American–British
KEGG: Kyoto Encyclopedia of Genes and Genomes
OS: overall survival
PB: peripheral blood
WBC: white blood cell
